# Interleukin-33 and RANK-L Interplay in the Alveolar Bone Loss Associated to Periodontitis

**DOI:** 10.1371/journal.pone.0168080

**Published:** 2016-12-19

**Authors:** Olivier Lapérine, Alexandra Cloitre, Jocelyne Caillon, Olivier Huck, Isaac Maximiliano Bugueno, Paul Pilet, Sophie Sourice, Elodie Le Tilly, Gaby Palmer, Jean-Luc Davideau, Valérie Geoffroy, Jérôme Guicheux, Sarah Beck-Cormier, Philippe Lesclous

**Affiliations:** 1 INSERM, U791, LIOAD, Nantes, France; 2 Université de Nantes, UMR-S 791, LIOAD, Nantes, France; 3 UFR Odontologie, Nantes, France; 4 ONIRIS, UMR-S 791, LIOAD, Nantes, France; 5 EA 3826 Thérapeutiques cliniques et expérimentales des infections, Nantes, France; 6 INSERM, U1109 Osteoarticular & Dental Regenerative Nanomedicine, Fédération de Médecine Translationnelle de Strasbourg (FMTS), Strasbourg, France; 7 Département de Parodontologie, Faculté de Chirurgie Dentaire, Université de Strasbourg, Strasbourg, France; 8 Division of Rheumatology, Department of Internal Medicine Specialties, University Hospitals of Geneva, Geneva, Switzerland; 9 Department of Pathology-Immunology, University of Geneva, School of Medicine, Geneva, Switzerland; 10 INSERM U1132 BIOSCAR, Hôpital Lariboisière, Paris, France; 11 Université Paris Diderot, Sorbonne Paris Cité, Paris, France; Medical University of South Carolina, UNITED STATES

## Abstract

**Introduction:**

Chronic Periodontitis (CP) is an inflammatory disease of bacterial origin that results in alveolar bone destruction. *Porphyromonas gingivalis* (*Pg*), one of the main periopathogens, initiates an inflammatory cascade by host immune cells thereby increasing recruitment and activity of osteoclasts, the bone resorbing cells, through enhanced production of the crucial osteoclastogenic factor, RANK-L. Antibodies directed against some cytokines (IL-1β, IL-6 and TNF-α) failed to exhibit convincing therapeutic effect in CP. It has been suggested that IL-33, could be of interest in CP.

**Objective:**

the present study aims to analyze whether and how IL-33 and RANK-L and/or their interplay are involved in the bone destruction associated to CP.

**Material and Methods:**

mRNAs and protein expressions of IL-33 and RANK-L were analyzed in healthy and CP human gingival samples by immunohistochemistry (IHC) and RT-qPCR. Murine experimental periodontitis (EP) was induced using *Pg* infected ligature and *Pg* free ligature around the first maxillary molar. Alveolar bone loss was recorded by μCT. Mouse gingival explants were stimulated for 24 hours with IL-33 and RANK-L mRNA expression investigated by RT-qPCR. Human oral epithelial cells were infected by *Pg* for 6, 12; 24 hours and IL-33 and RANK-L mRNA expressions were analyzed by RT-qPCR.

**Results:**

IL-33 is overexpressed in gingival epithelial cells in human affected by CP as in the murine EP. In human as in murine gingival cells, RANK-L was independently induced by *Pg* and IL-33. We also showed that the Pg-dependent RANK-L expression in gingival epithelial cells occured earlier than that of IL-33.

**Conclusion:**

Our results evidence that IL-33 overexpression in gingival epithelial cells is associated with CP and may trigger RANK-L expression in addition to a direct effect of *Pg*. Finally, IL-33 may act as an extracellular alarmin (danger signal) showing proinflammatory properties in CP perpetuating bone resorption induced by *Pg* infection.

## Introduction

Periodontitis refers to an inflammatory disease of bacterial origin that affects tissues surrounding and supporting the tooth ie gingiva, periodontal ligament, cementum and alveolar bone. The hallmark of periodontitis is a destruction of alveolar bone resulting ultimately in extended tooth loss and oral disability [1]. Growing evidence indicates that periodontitis is highly prevalent in adult population according to a 2012 US survey underlining that 47% of this population is affected by periodontitis, 8.5% in its severe form. This rate increased to 64% in aging persons older than 65 years and is expected to increase with age [2]. Periodontitis is a major health challenge particularly affecting the elderly where the disease is the primary cause of tooth loss but also because periodontitis interplays with systemic health, particularly by increasing the patients’ risk and morbidity for atherosclerosis, rheumatoid arthritis (RA) and diabetes mellitus [3, 4]

An anaerobic bacterium, *Porphyromonas gingivalis* (*Pg*), is traditionally considered a major causative agent of periodontitis, based on its virulence properties and strong association with diseased sites. However, *Pg*-induced periodontitis required the presence of commensal microbiota for the onset of periodontitis [5]. In a recently proposed definition, periodontitis may result not from individual pathogens, but rather from polymicrobial synergy and dysbiosis, a condition characterized by an imbalance in the relative abundance or influence of species within a microbial community [3].

It is now well acknowledged that the presence of bacterial species is necessary but not sufficient for the onset and progression of periodontitis. The recognition of microbial components as “danger signals” by host immune cells and subsequent production of inflammatory mediators is an essential step in periodontitis pathogenesis [6]. Production of pro-inflammatory cytokines (including IL-1β, IL-6, and TNF-α) by resident and recruited inflammatory cells acting synergistically seemed to be of particular importance in this process. Indeed, these cytokines increase the recruitment and activity of the bone resorbing cells, the osteoclasts, through enhanced production of a crucial osteoclastogenic factor, the Receptor Activator of Nuclear Factor κ B Ligand (RANK-L) and favor bone destruction [7]. However antibodies directed against these 3 cytokines did not exhibit a convincing therapeutic effect in periodontitis, thereby strongly suggesting that others mediators could be involved in the pathogenesis of this disease [8]. Recently, it has been suggested that among inflammatory mediators involved in chronic periodontitis (CP), a member of the IL-1 family, IL-33, could play a role in the initiation and the progression of CP [9].

IL-33 has been described to regulate innate and adaptive immunity [10]. IL-33 is constitutively expressed as a nuclear factor in many cell types including epithelial cells, fibroblasts and endothelial cells. Sequestrated in the nucleus of these cells, IL-33 is considered as an endogenous molecule that allows the maintenance of the crucial transcription factor NFκB and thus reduces the expression of genes encoding for proinflammatory cytokines thereby ensuring tissue homeostasis. When released in the extracellular medium upon cell damage or stress, IL-33 acts as an alarmin (also known as danger signal) showing proinflammatory properties [11–13]. IL-33 acts through its ST2 receptor, widely expressed on many cell types of innate and adaptive immunity such as mastocytes, Th2 lymphocytes and B cells leading to pro-inflammatory cytokines production [10, 14, 15]. Interestingly, some studies have highlighted a potential role for IL-33 in the regulation of inflammatory process related to RA, a disease sharing many immunopathological similarities with periodontitis [16]. In mice, inhibition of IL-33 signaling with a soluble isoform of ST2 (sST2) acting as a decoy receptor or deletion of ST2 has been shown to attenuate the severity of induced arthritis in part by blocking RANK-L expression [17]. However, ST2 deletion in a mouse model of K/BxN serum transfer-induced arthritis did not protect animals from inflammation and bone resorption [18]. These contradictory results suggest that the role of IL-33 in inflammatory process leading to bone destruction is highly questionable, probably local or even focal and timely regulated depending of the inflammatory microenvironment.

The potential role for IL-33 in the onset and progression of periodontal disease is emerging and mostly considered as a proinflammatory factor. In a rat model of ligature induced-periodontitis, IL-33 expression was upregulated concomitantly to RANK-L [19]. In human, high IL-33 overexpression was recorded in the gingiva of patients affected by CP and may act as a triggering factor for the recruitment of B and T lymphocytes expressing RANK-L [20]. But in gingival crevicular fluid (an inflammatory exudate collected in the periodontal pocket) conflicting results regarding IL-33 levels have been reported in patients affected by CP [21–23]. Interestingly, *Pg* has been described to upregulate IL-33 mRNA expression in gingival epithelial cells through the PAR-2 signaling pathway [24]. But to date, it is still unclear whether bacterial or pro-inflammatory stimulus first triggers IL-33 expression in the gingival tissue. Contrasting with its positive effect on RANK-L expression, IL-33 has also been reported to inhibit osteoclast differentiation *in vitro*, suggesting a protective effect on bone [25–27]. Taken together, these data suggest a multiple and contrasting role for IL-33 during inflammatory diseases associated to bone resorption such as CP that need to be clarified.

In periodontitis, multiple sources of RANK-L have been proposed such as osteoblasts, B and T lymphocytes or epithelial cells [28, 29]. Interestingly, gingival epithelial cells, the first-line cell population in contact with periopathogens, can produce various pro-inflammatory cytokines and basal level of RANK-L to support osteoclastogenesis [30–32]. Malcolm et al described an increased expression of IL-33 in gingival epithelial cells from patient affected by CP. However, the contribution of this cell type cell to the onset and the progression of the disease through RANK-L expression have not been elucidated.

The present study aims to analyze whether and how IL-33 and RANK-L and/or their interplay are involved in the bone destruction associated to CP.

We showed in this study that IL-33 is overexpressed in gingival epithelial cells in human affected by CP as in a murine model of experimental periodontitis (EP). Moreover, in human as in murine gingival cells, we showed that RANK-L expression can be independently induced by *Pg* and IL-33. These results strengthen a potent role for IL-33 in the pathogenesis of periodontitis and suggest that both *Pg* and IL-33 induce overexpression of RANK-L and subsequently increase alveolar bone loss.

## Materials and Methods

### Patients

The human study protocol, consent forms and consent procedure were reviewed and approved by the Institutional Medical Ethic Comittee of the Universitary Hospital of Nantes (SVTO:DC-2011-1399). All patients provided their written consent to participate to this study. Detailed medical and periodontal histories of each patient were recorded (Table 1). According to the American Association of Periodontology, criteria for CP, were i) probing pocket depth > 5 mm, ii) clinical attachment level > 3 mm and iii) bleeding on probing periodontal pockets [33].

**Table 1:** Characteristics of the patients.

Variables	Chronic periodontitic patients		Healthy patients	
	Number	%	Number	%
**Gender**				
Male	10	76.9	3	33.3
Female	3	23.1	6	66.7
**Age (years)**	48.3 ± 8.5		20.33 ± 3.6	
**Tobacco**				
User	8	61.5	4	44.4
Non-user	5	38.5	5	55.6
**Probing pocket depth (mm)**	5.3 ± 1.9		NA*	
**Clinical attachment level (mm)**	7.8 ± 1.9		NA*	
**Bleeding on probing**	13	100	0	0

* NA: Not applicable

Variables: mean values [SD]

In patients diagnosed for CP (n = 13), gingival samples between 5 to 8 mm sides and 3 to 5 mm deep were obtained during the surgical treatment, an open flap debridement combined with dental extractions and gingival regularization. Healthy gingival samples were harvested from patients (n = 9) without any periodontal diseases undergoing oral surgical procedures needing gingival regularization such as extraction of impacted third molars. No patients had taken any anti-inflammatory medication for 2 weeks before surgical procedures. The gingival samples were used for RT-qPCR and/or histology.

### Murine model of alveolar bone loss

Animal studies were approved by the Ethic Committee for Animal Experiment of Pays de la Loire (CEEA 2012.187). Mice were housed in specific-pathogen-free facilities, and under light- (12h light/dark cycle), temperature- (22–25°C) and humidity- (50–60%) controlled conditions. To avoid any gingival effect of estrogens, we only used 12 week-old male CD1 Swiss mice (Janvier). All animals were fed with regular diet. A wash-out period was first performed in which mice were treated with water diluted sulfomethoxazole-trimethoprim 0.2mg/mL (Roche) for ten days followed by a 3 days antibiotic-free period [34]. For periodontal procedures, mice were anesthetized with intraperitoneal injection of ketamine (80 mg/kg, Imalgene 1000, Merial) and xylazine (10mg/kg, Rompun 2%, Bayer). *Pg*-infected and control mice were placed in separated cages and kept in the same environment. Mice were monitored in order to evaluate pain stress, and weighed on a daily basis.

**Fig 1:**
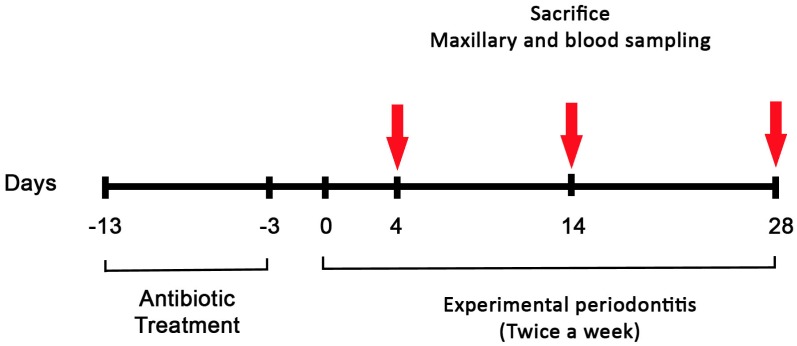
Study design of the murine model of experimental periodontitis.

A time course experiment was conducted with 90 animals randomly distributed in 9 experimental groups of 10 mice. EP was induced by placement of a 6.0 silk ligature soaked wih *Pg* (*Pg* Lig) or not (L) in the gingival crevice (so called sulcus) around the first maxillary molar for 4, 14 or 28 days. This procedure was repeated twice a week (Fig 1). Control mice received a slight incision into the sulcular epithelium to mimic the ligature placement (Sham). At every time periods, mice were sacrificed by cardiac exsanguination under the same anesthesia procedure as previously described for periodontal procedure.

Blood samples were collected by cardiac puncture and sera were prepared by centrifugation at 2000xg for 2 minutes.

Micro-computed tomography and histological analyses were performed on sampled maxilla fixed in 4% paraformaldehyde at 4°C for 24 hours.

### Bacterial and strain culture

*Pg* (ATCC 33277) was cultured at 37°C on Shaedler agar plated with sheep blood (BD), in an oxygen-free atmosphere. After 10 days of culture, *Pg* colonies were selected and resuspended in brain-heart broth at 10^9^ CFU/mL for ligature placement or at 10^5^ CFU/mL for *Pg* injection. A 6–0 silk thread was placed in the suspension 24 hours before EP procedure.

### Gingival explant culture

Palatal mucosa from 3 C57BL/6J mice was harvested and placed connective face in contact with plate for 1 hour to allow cell adhesion. Explants were then cultured in DMEM supplemented with 10% FCS, 50U/ml Penicillin and 50 mg/ml Streptomycin for 24 hours. They were then stimulated with 100ng/mL of recombinant murine IL-33 (3626-ML-010, R&D) in serum-free DMEM supplemented with 50U/ml Penicillin and 50 mg/ml Streptomycin for 24 hours. Three independent experiments were performed.

### Micro-computed tomography (μCT)

Two and three dimensional analyses of the maxilla were performed using a Skyscan 1272 (Skyscan). Acquisition parameters were 60 kV, 80 mA, 0.25-mm aluminium filter. The NRecon software (Skyscan) was used for reconstruction. For the two-dimensional analyses, distance between the palatal cementum-enamel junction of the first molar and the alveolar bone crest (CEJ-ABC) was measured to assess alveolar bone loss on the coronal plan. Five sections per animal were analyzed when radicular canal for each tooth were fully visible. For the three-dimensional analyses, bone mineral volume/tissue volume (MBV/TV) was assessed in an elliptical region of interest (ROI) between CEJ and the root apices of the first molar in the axial plan. The fraction of MBV/TV was calculated using CTan software and data are presented as percentage of ROI area. Evaluations were performed by two independent operators.

### Immunohistochemistry (IHC)

Fixed maxilla were decalcified in EDTA 0.5M at 4°C for three weeks. Human and mouse samples were then dehydrated and embedded in paraffin. IHC were performed on 4 μm-thick sections. Antigens were retrieved by boiling slides for 30 minutes in citrate buffer pH 6. Sections were then incubated in blocking solution (S2022, Dako) for 45 minutes at RT. Incubation with primary antibodies was performed in the blocking solution at 4°C over-night. Sections were rinsed three times (0.05% tween in PBS) and endogenous peroxydase activity was quenched with 3% H2O2 in PBS for 20 minutes. Specific binding was detected using DAB (Dako). Sections were counterstained with Harris Hematoxilin, dehydrated and mounted in Eukitt. Primary and secondary antibodies used are: polyclonal goat anti-IL-33 (AF3626 and AF3625; R&D systems), polyclonal goat anti-RANK-L (sc7628, clone N-19, 1/100; Santa Cruz) and monoclonal rat anti-human CD3 (clone CD3-12, 1/200; AbD Serotec), anti-goat HRP antibody (1:500; sc-2961, Santa Cruz Biotechnology), anti-rat HRP antibody (1:500, Jackson Immunoresearch inc,). Specificity of IL-33 antibody was assessed using maxilla samples from IL-33 KO mice. Automated whole slide imaging was performed using the NanoZoomer 2.0 (Hamamatsu). All analyses were performed in multiple randomly selected high-power microscopic fields (magnification x200). Staining quantification on four sections per sample was performed using Fiji software (NIH).

### RT-qPCR

Total RNA was extracted from human gingival samples and mouse gingival explants using the Nucleospin RNA isolation kit (Macherey Nagel). cDNA were synthesized using SuperScript III First-strand Synthesis System (Life Technologies). Twenty nanograms of cDNA were used to assess mRNA expression by using TaqMan gene expression assays (Life technologies). The probe and primer sets for Human *IL33* (Hs01125943_m1), RANK-L (*Tnfsf11*, Hs00243522_m1), *TNF-α* (Hs01113624_g1), *IL-6* (Hs00985639_m1) and mouse RANK-L (*Tnfsf11*, Mm01313943_m1) and the normalizers human *Ppia* (Hs99999904_m1) and mouse *GusB* (Mm01197698_m1) were obtained from Applied Biosystems. Measurements were performed in triplicate. Relative quantification was determined using the Biorad CFX manager software (Biorad).

### Enzyme-linked immunosorbent assay (ELISA)

Serum IL-33 concentrations were measured according to manufacturer’s instructions (Kit duoset DY3626-05 ELISA R&D). Briefly, plates were coated with a goat anti-mouse IL-33 antibody overnight. Then samples were incubated during 1 hour. A biotinylated goat anti-mouse IL-33 antibody was added for 2 hours. Streptavidin-HRP was added for 20 minutes and the reaction was visualized by the addition of 50 μl chromogenic substrate (TMB) for 30 minutes. The reaction was stopped with 100 μl H2SO4 and absorbance at 450 nm was measured using an ELISA plate reader (Victor3, Wallac 1420, PerkinElmer). All procedures were performed at room temperature.

### Cell culture

Oral epithelial cells (OECs) derived from Human oral keratinocyte cell line TERT-2 OKF-6 (BWH Cell Culture and Microscopy Core) were cultured in defined Keratinocyte-SFM basal medium (KSFM) supplemented with growth supplements (Invitrogen).

#### Infection and stimulation of OECs

Twenty-four hours before infection with *Pg*, 3 x 10^5^ cells per well were seeded in 24-well plate. On the day of the infection or stimulation, cells were washed with PBS and medium without antibiotics containing *Pg* (MOI 100:1 or MOI 10:1) was added for 24 hours.

#### Quantitative RT-qPCR analysis

RT-qPCR was performed to quantify RNA expression. PCR amplification and analysis were achieved using the CFX Connect ^™^ Real-Time PCR Detection System (Bio-Rad). Amplification was performed using iTaq Universal SYBR Green Supermix. Beta-actin was used as endogenous control in the samples. Primers sequences related to *IL-33* (5’-GGTGTTACTGAGTTATATGAG-3’, 3’-GGAGCTCCACAGAGTGTTCCTTG-5’) and RANKL (*Tnfsf11)* (5’-GCCAGTGGGAGATGTTAG -3’, 3’- CCCTTTTGAACGTCGATT-5’) were synthesized by Eurofins (Ebersberg).) Relative quantification was determined using the Biorad CFX manager software (Biorad). Three separate sets of experiments were performed for each procedure.

### Statistical analysis

Data were compared using non-parametric tests (Kruskal-Wallis test followed, if significant, by group comparisons with the Mann-Whitney test or the unpaired student t-test. Differences were considered significant if p ≤ 0.05. Results are given as means ± SEM or means ± SD for patient’s characteristics.

## Results

### IL-33 and RANK-L are overexpressed in human chronic periodontitis

**Fig 2:**
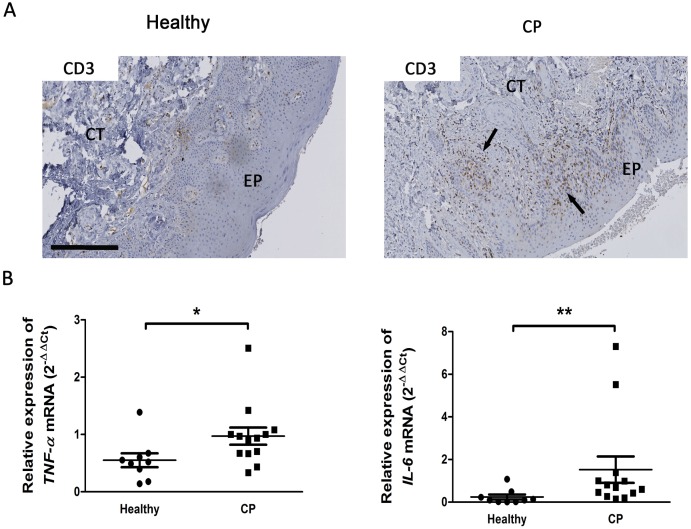
Characterization of human gingival samples in healthy and patients affected by chronic periodontitis. A. Samples were stained for the T lymphocytes marker CD3 (arrows). Sections were counterstained with Harris Hematoxylin staining. EP: Epithelium; CT: Connective Tissue. B. TNF-α and IL-6 expression in healthy and CP patients were measured by RT-qPCR. Data are shown as mean ± SD. Healthy samples n = 9; chronic periodontitis samples n = 13. Bar = 250μm. *p<0.05, **p<0.01.

First, we analyzed clinical parameters to determine the inflammatory status of CP samples (Table 1) and expression of well documented inflammatory markers such as CD3, IL-6 and TNF-α by IHC. We observed a strong positive CD3 immunostaining in CP samples both in connective tissue and in gingival epithelium (Fig 2A). Expression mRNA of the pro-inflammatory cytokines TNF-α and IL-6 was investigated by RT-qPCR. Significant overexpression of mRNA encoding for both cytokines was recorded in CP compared to healthy samples (p<0.05) (Fig 2B). No differences were recorded according to the gender or the tobacco habit.

**Fig 3:**
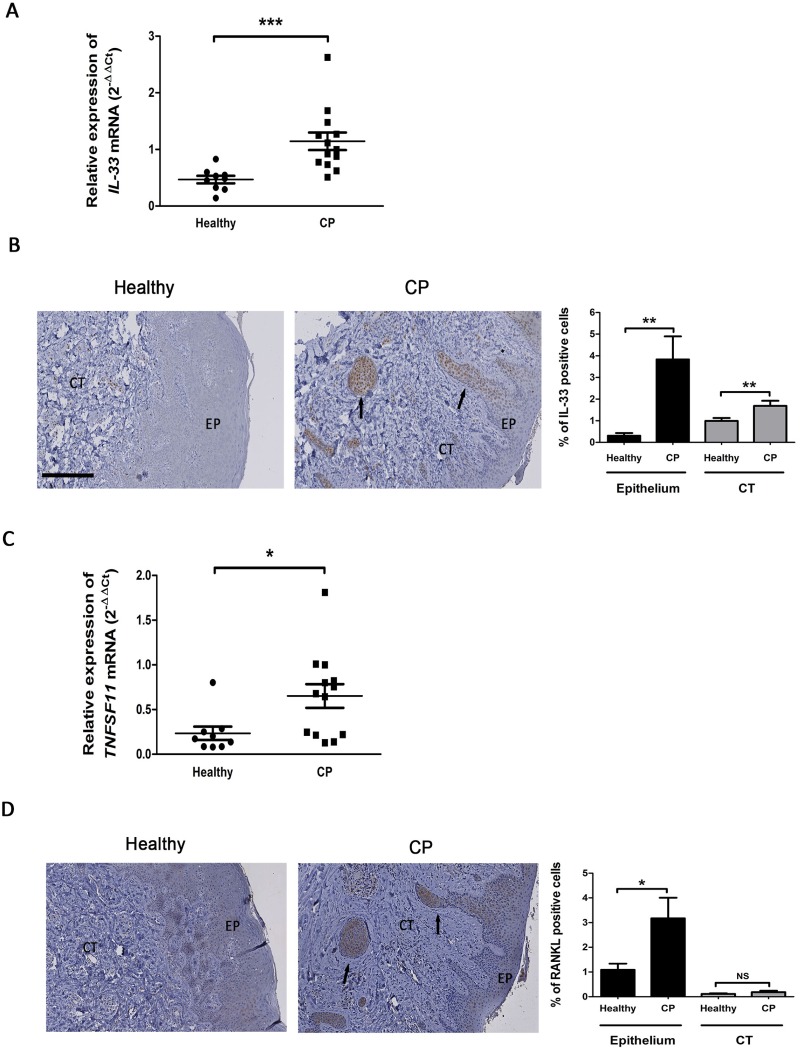
IL-33 and RANK-L expressions in gingival samples of healthy and patients affected by chronic periodontitis. A. mRNA encoding for IL-33 was quantified by RT-qPCR. B. Healthy and CP gingival samples were immunostained for IL-33 (arrows). The percentage of cells positive for IL-33 was quantified using Fiji software and defined as a percentage of DAB positive staining area per region of interest. C. mRNA encoding for RANK-L was quantified by RT-qPCR. D. Healthy and CP gingival samples were immunostained for RANK-L (arrows). The percentage of cells positive for RANK-L was quantified using Fiji software and defined as a percentage of DAB positive staining area per region of interest. EP: Epithelium; CT: Connective Tissue. Data are shown as mean ± SEM. Healthy samples n = 9; Chronic periodontitis samples (CP) n = 13. Bar = 250μm. *p<0.05; **p<0.01; ***p<0.001.

We then determine whether IL-33 and RANK-L can also be overexpressed in CP gingival samples when compared to healthy gingival samples. As indicated in Fig 3A, IL-33 mRNA was significantly increased in CP samples (p<0.001). Immunolabeling for IL-33 was also increased in CP compared to healthy samples (p<0.001) (Fig 3B). Interestingly, quantification of IL-33 positives cells revealed a drastic 8-fold increase in the gingival epithelium and a slighter but significant 1.5-fold increase in the connective tissue of CP samples (p<0.01). As expected, transcript coding for RANK-L was significantly higher in CP samples (p<0.05) (Fig 3C) and IHC revealed a 3-fold RANK-L overexpression in CP samples but only in the epithelial compartment (p<0.05) (Fig 3D). Interestingly, this epithelial RANK-L overexpression appeared in parallel with that of IL-33.

### Bone loss is associated with IL-33 overexpression in a murine model of experimental periodontitis

**Fig 4:**
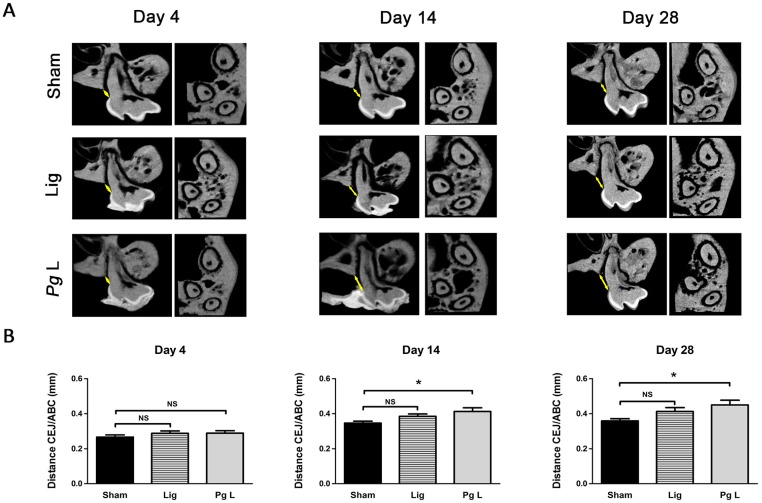
Time-course of alveolar bone loss in the ligature-induced murine model of experimental periodontitis. CD1 Swiss mice (n = 90) were subjected to experimental periodontitis for 4, 14 and 28 days. At each time point, animals were sacrificed and maxillary samples were harvested. A. After 4, 14 and 28 days, μCT analysis was performed. Longitudinal sections through the middle of the palatal root of the first maxillary molar (left images) and transversal sections from the apices of the three roots of the first maxillary molar to the summit of the alveolar bone crest (right images) are presented for each time points. B. Alveolar bone loss was assessed using 2D μCT. At each time point, data of ligatured groups (Lig and *Pg* L) were compared to their respective Sham groups. Data are shown as means ± SEM. * p<0.05.

**Fig 5:**
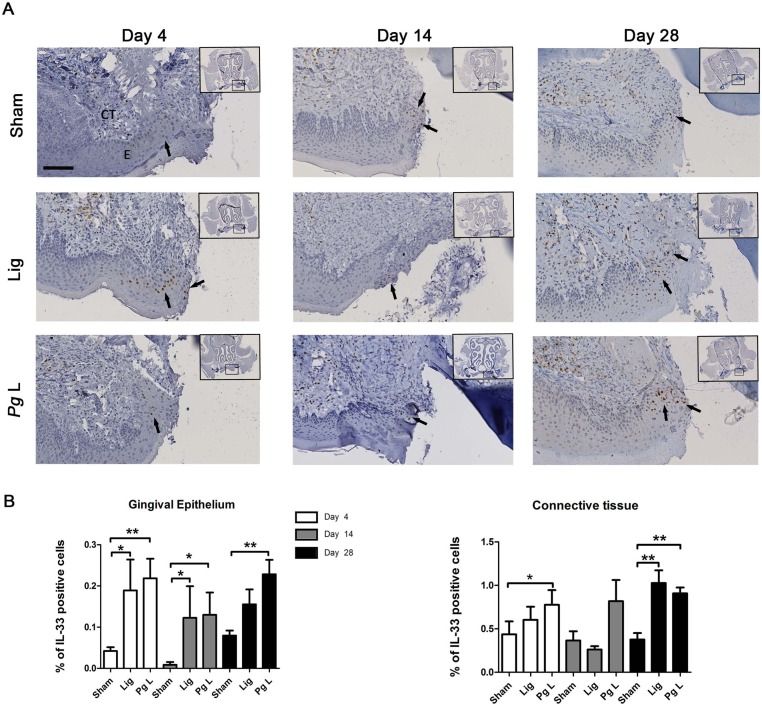
Time-course of IL-33 expression in the ligature-induced murine model of experimental periodontitis. A. IL-33 expression was assessed by IHC and sections were counterstained with Harris Hematoxylin staining (arrows). B. The percentage of IL-33 was quantified in gingival epithelium and in connective tissue using Fiji software and defined as a percentage of DAB positive staining area per region of interest. At each time point, data of ligatured groups (Lig and *Pg* L) were compared to their respective Sham groups. EP: Epithelium, CT: Connective tissue. Data are shown as means ± SEM. * p<0.05; **p<0.01. Scale bar = 100μm.

We used a murine model of EP to determine whether IL-33 may be associated to the alveolar bone loss occurring in CP in a time course study with three experimental groups “*Pg* ligature” (*Pg* L), “Ligature” (Lig) and “Sham” mimicking the ligature apposition. Alveolar bone loss induced by *Pg* ligature was significant as early as 14 days following surgery and persisting until day 28 when compared to sham group (p<0.05) (Fig 4A and 4B). *Pg* free ligature failed to induce significant alveolar bone loss compared to sham group (Fig 4A and 4B). After having confirmed that *Pg* ligature induced significant alveolar bone loss, we further addressed if IL-33 may be implicated in this process. Immunostaining revealed an increase of IL-33 positive cells in the connective tissue of *Pg* ligature group when compared to sham group 4 days after surgery (p<0.05). We also recorded such higher IL-33 positive cells in ligature and *Pg* ligature groups when compared to sham at 28 days (p<0.01) (Fig 5A and 5B). Interestingly, increases of IL-33 positive cells were also recorded in the gingival epithelium of the ligature and *Pg* ligature groups at 4 and 14 days after surgery (p<0.05 and p<0.01 respectively at 4 days; p<0.05 and p<0.05 respectively at 14 days) (Fig 5A and 5B). Significant increase of IL-33 positive cells was sustained in *Pg* ligature group at 28 days (p<0.001) but not in ligature group compared to sham at this time, indicating that *Pg* is necessary for a continuous expression of IL-33 in gingival cells (Fig 5A and 5B). Globally, our data indicates that IL-33 increased in mice in epithelial and in connective compartments, as observed in human samples, before the onset of alveolar bone loss and that *Pg* sustained IL-33 expression in gingival epithelial cells during EP.

### IL-33 increased RANK-L expression in mouse gingival explants

**Fig 6:**
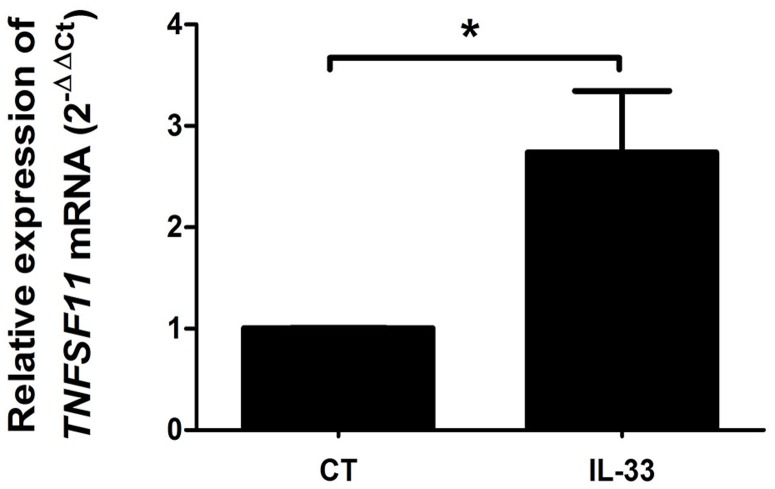
IL-33 induced RANK-L expression in mouse gingival explants. Explants from palatal mucosa of C57BL/6 mice were culture overnight at 37°C. These explants were then stimulated with 100ng/mL of recombinant murine IL-33 for 24 hours. Total tissue RNA was extracted and RANK-L transcript was quantified by RT-qPCR. Three separate experiments were performed. Data are shown are means ± SEM. *p<0.05.

To further analyze whether IL-33 can be involved in the increased alveolar bone loss associated to periodontitis, we used mouse gingival explants treated with IL-33 for 24 hours. RT-qPCR analyses evidenced a significant increase of RANK-L mRNA expression 24 hours after IL-33 stimulation (p<0.05) (Fig 6). This demonstrates the ability of IL-33 to induce RANK-L expression in murine gingival cells.

### *Pg* infection resulted in an overexpression of RANK-L mRNA but was a weak inducer of IL-33 mRNA in human oral epithelial cells

**Fig 7:**
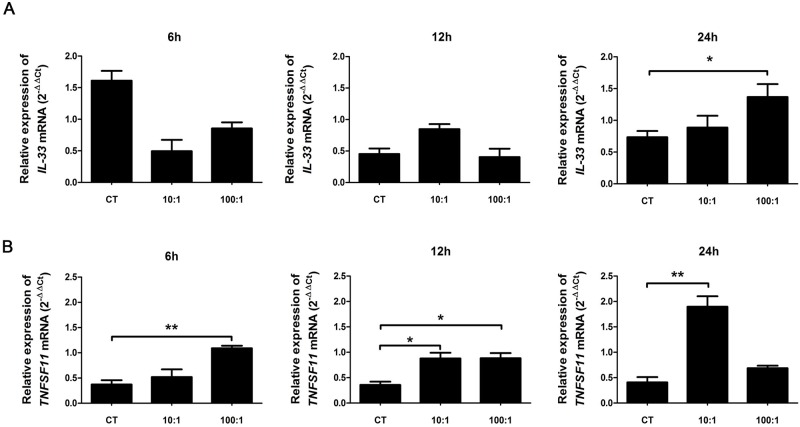
*Pg* infection increased the expression of RANK-L and IL-33 mRNAs in human oral epithelial cells. Human oral epithelial cells (OKF6/TERT2) were cultured with *Pg* at 10:1 or 100:1 MOI for 6, 12 or 24 hours. mRNAs encoding for IL-33 (A) and RANK-L ((B) were quantified by RT-qPCR. Three separate sets of experiment were performed. Data are shown as mean ± SEM. *p<0.05; **p<0.01.

We also investigated whether *Pg* could be the triggering factor to induce IL-33 and RANK-L in human OECs. OECs were infected by *Pg* for 6, 12 and 24 hours at MOI of 10:1 or 100:1. RT-qPCR analysis revealed that IL-33 mRNA expression was only significantly increased at MOI of 100:1 24 hours after *Pg* infection (p<0.05) (Fig 7A). Expression of RANK-L was stable throughout the experiment in control cells. After *Pg* infection, this RANK-L expression increased from 6 to 24 hours at MOI10:1 but was quite stable at MOI 100:1. A significant overexpression of RANK-L was evidenced for each time of infection (6 hours: MOI 100:1, p<0.01; 12 hours: MOI 100:1 and 10:1, p<0.05; 24 hours: MOI 10:1, p<0.01) (Fig 7B). These results suggest that *Pg* may trigger RANK-L expression in gingival epithelial cells but is a weak inducer of IL-33 expression.

## Discussion

In this study, we demonstrated that gingival epithelial cells could play an undescribed role in the alveolar bone loss associated to periodontal disease throughout RANK-L production. We also evidenced that RANK-L overexpression could be mediated by *Pg* and/or through IL-33 expression in gingival epithelial cells.

IL-33 protein expression was increased in gingival epithelial cells both in human and mouse. IL-33 expression in CP in human has recently been described by Malcolm et al. in gingival epithelial cells and our results confirmed this observation [20]. This is of particular interest because differences of IL-33 expression in skin have been reported between species [35]. Notably in human and porcine keratinocytes, IL-33 expression is low but rapidly induced after skin wounding whereas it is the opposite in mice. Here, we evidenced in a murine model of EP induced by the apposition of a *Pg*-soaked ligature that IL-33 expression was increased in gingival epithelial cells similarly as in human CP. This sustains the reliability of this experimental model to mimic the alveolar bone loss related to periodontitis in human and so, to investigate the potent role of IL-33 in the inflammatory cascade associated to the CP pathogenesis.

We have confirmed that IL-33 expression is increased in human gingiva from CP patients and we have also evidenced this increase in a ligature-induced model of EP. Two major models of EP are described in the literature. The oral gavage model is useful for addressing a wide variety of hypotheses related to periodontal pathogenesis, ranging from the role of the host response to virulence traits of pathogens and to the interconnections of those factors with systemic parameters. The silk ligature model is more relevant to investigate mechanistic and inflammatory aspects of the host immune response to bacterial stimulus and to identify potent therapeutic targets of particular interest in this inflammatory cascade [36]. This experimental model is also the most appropriate as it mimics the start point of periodontitis, a local gingival infection where oral epithelial cells are the first-line defense. Indeed, in our study, the inflammatory process seems strictly localized to the stimulated area because no alveolar bone loss was recorded elsewhere. We didn’t evidence any increase in serum concentration of IL-33 in mice subjected to ligatures impregnated or not with *Pg*. This hardly suggests that IL-33 is not involved in a systemic inflammatory process but rather in a gingival localized one to possibly trigger alveolar bone loss.

The first key factor for the onset of periodontitis is the presence of pathogen species such as *Pg*. Increased expression of inflammatory cytokines and particularly IL-33 has been previously described *in vitro* after *Pg* infection of gingival epithelial cells [24, 31]. We also recorded such increase 24 hours after *Pg* infection of human epithelial cells. However, as we also recorded an increased IL-33 expression *in vivo* independently of *Pg* infection, it is tempting to speculate that the ligature apposition is an inflammatory stimulus sufficient to induce the increase of IL-33 expression in gingival epithelial cells, directly or throughout other pro-inflammatory cytokines such as TNF-α or IFN-γ [35, 37]. Consistently, we have also observed an IL-33 expression in Sham animals subjected to a slight incision suggesting that this mechanical stimulus may trigger the production of IL-33 in gingival epithelial cell and that *Pg* may be not the major factor needed for IL-33 expression.

In CP, increased osteoclast activity is related to increased production of RANK-L. Several sources of RANK-L have been described notably B and T lymphocytes [28]. In our study, we showed a concomitant increase in CD3 and RANK-L positive cells and in RANK-L expression in gingival epithelial cells of patients affected by CP. RANK-L-producing lymphocytes are recruited towards inflammatory sites following *Pg* infection [38]. *Pg* can also stimulate RANK-L production by other cells such as bone alveolar osteoblasts and periodontal ligament fibroblasts notably via TLR2 signaling [39]. Mice deficient for TLR2 were protected from *Pg*-induced alveolar bone loss indicating that *Pg* is a crucial factor for the increase in RANK-L expression observed in CP [39]. Gingival epithelial cells, the first cells in contact with bacterial stimulus, are also able to produce RANK-L when stimulated by TNF-α [32]. In patients affected by CP, we recorded an increase in RANK-L expression in epithelial cells at the close vicinity of inflammatory sites, supporting a potential role to trigger the recruitment of osteoclast precursors, the osteoclast differentiation and activity as suggested by others [29].

IL-33-induced RANK-L expression in lymphocytes of the gingival tissue has been observed in EP induced by oral gavage and treatment with IL-33 [20]. We showed, using RT-qPCR quantification, that RANK-L was stimulated before IL-33 expression after *Pg* infection of gingival epithelial cells. This finding suggests that overexpression of RANK-L in CP is not primarily caused by IL-33 and that this cytokine may act as a secondary factor able to promote bone resorption through RANK-L after gingival infection by *Pg*. We can also hypothesize that IL-33 could be required for the perpetuation of alveolar bone loss in CP and we propose that alveolar bone loss related to CP is induced by the combination of *Pg* infection and IL-33.

EP applied to IL-33 knock-out mice will be needed to decipher the potent role of IL-33 in alveolar bone loss associated to CP. Taken together, these data highlight the ability of IL-33 to induce RANK-L expression in gingival cells and the interplay between both factors in CP.

## Conclusion

Our results provide evidences that IL-33 overexpression in gingival epithelial cells is associated with CP and can trigger RANK-L expression in addition to the direct effect of *Pg*. Finally, IL-33 may act as an extracellular alarmin showing proinflammatory properties in CP by perpetuating bone resorption induced by *Pg* infection.

## References

[1] Pihlstrom BL, Michalowicz BS, Johnson NW. Periodontal diseases. The Lancet. 2005;366(9499):1809–20. 10.1016/S0140-6736(05)67728-8 16298220

[2] Eke PI, Dye BA, Wei L, Thornton-Evans GO, Genco RJ, Cdc Periodontal Disease Surveillance workgroup: James Beck GDRP. Prevalence of periodontitis in adults in the United States: 2009 and 2010. Journal of dental research. 2012;91(10):914–20. 10.1177/0022034512457373 22935673

[3] Hajishengallis G. Periodontitis: from microbial immune subversion to systemic inflammation. Nature reviews Immunology. 2015;15(1):30–44. 10.1038/nri3785 25534621 PMC4276050

[4] Eke PI, Dye BA, Wei L, Slade GD, Thornton-Evans GO, Borgnakke WS, et al Update on Prevalence of Periodontitis in Adults in the United States: NHANES 2009 to 2012. Journal of periodontology. 2015;86(5):611–22. 10.1902/jop.2015.140520 25688694 PMC4460825

[5] Hajishengallis G, Liang S, Payne MA, Hashim A, Jotwani R, Eskan MA, et al Low-abundance biofilm species orchestrates inflammatory periodontal disease through the commensal microbiota and complement. Cell host & microbe. 2011;10(5):497–506. 22036469 10.1016/j.chom.2011.10.006 PMC3221781

[6] Graves DT, Oates T, Garlet GP. Review of osteoimmunology and the host response in endodontic and periodontal lesions. Journal of oral microbiology. 2011;3:5304–19. 10.3402/jom.v3i0.5304 PMC3087239 21547019

[7] Hienz SA, Paliwal S, Ivanovski S. Mechanisms of Bone Resorption in Periodontitis. Journal of immunology research. 2015;2015:615486–95. 10.1155/2015/615486 26065002 PMC4433701

[8] Preshaw PM, Taylor JJ. How has research into cytokine interactions and their role in driving immune responses impacted our understanding of periodontitis? Journal of clinical periodontology. 2011;38 Suppl 11:60–84. 21323705 10.1111/j.1600-051X.2010.01671.x

[9] da Luz FA, Oliveira AP, Borges D, Brigido PC, Silva MJ. The physiopathological role of IL-33: new highlights in bone biology and a proposed role in periodontal disease. Mediators of inflammation. 2014;2014:342410–7. 10.1155/2014/342410 24692848 PMC3945897

[10] Schmitz J, Owyang A, Oldham E, Song Y, Murphy E, McClanahan TK, et al IL-33, an interleukin-1-like cytokine that signals via the IL-1 receptor-related protein ST2 and induces T helper type 2-associated cytokines. Immunity. 2005;23(5):479–90. 10.1016/j.immuni.2005.09.015 16286016

[11] Moussion C, Ortega N, Girard JP. The IL-1-like cytokine IL-33 is constitutively expressed in the nucleus of endothelial cells and epithelial cells in vivo: a novel 'alarmin'? PLoS One. 2008;3(10):e3331 10.1371/journal.pone.0003331 18836528 PMC2556082

[12] Byers DE, Alexander-Brett J, Patel AC, Agapov E, Dang-Vu G, Jin X, et al Long-term IL-33-producing epithelial progenitor cells in chronic obstructive lung disease. The Journal of clinical investigation. 2013;123(9):3967–82. 10.1172/JCI65570 23945235 PMC3754239

[13] Carriere V, Roussel L, Ortega N, Lacorre DA, Americh L, Aguilar L, et al IL-33, the IL-1-like cytokine ligand for ST2 receptor, is a chromatin-associated nuclear factor in vivo. Proceedings of the National Academy of Sciences of the United States of America. 2007;104(1):282–7. 10.1073/pnas.0606854104 17185418 PMC1765450

[14] Moulin D, Donze O, Talabot-Ayer D, Mezin F, Palmer G, Gabay C. Interleukin (IL)-33 induces the release of pro-inflammatory mediators by mast cells. Cytokine. 2007;40(3):216–25. 10.1016/j.cyto.2007.09.013 18023358

[15] Komai-Koma M, Gilchrist DS, McKenzie AN, Goodyear CS, Xu D, Liew FY. IL-33 activates B1 cells and exacerbates contact sensitivity. J Immunol. 2011;186(4):2584–91. 10.4049/jimmunol.1002103 21239718

[16] Liew FY, Pitman NI, McInnes IB. Disease-associated functions of IL-33: the new kid in the IL-1 family. Nature reviews Immunology. 2010;10(2):103–10. 10.1038/nri2692 20081870

[17] Palmer G, Talabot-Ayer D, Lamacchia C, Toy D, Seemayer CA, Viatte S, et al Inhibition of interleukin-33 signaling attenuates the severity of experimental arthritis. Arthritis and rheumatism. 2009;60(3):738–49. 10.1002/art.24305 19248109

[18] Martin P, Talabot-Ayer D, Seemayer CA, Vigne S, Lamacchia C, Rodriguez E, et al Disease severity in K/BxN serum transfer-induced arthritis is not affected by IL-33 deficiency. Arthritis research & therapy. 2013;15(1):R13. 23324173 10.1186/ar4143 PMC3672723

[19] Koseoglu S, Hatipoglu M, Saglam M, Enhos S, Esen HH. Interleukin-33 could play an important role in the pathogenesis of periodontitis. Journal of periodontal research. 2015;50(4):525–34. 10.1111/jre.12235 25266494

[20] Malcolm J, Awang RA, Oliver-Bell J, Butcher JP, Campbell L, Adrados Planell A, et al IL-33 Exacerbates Periodontal Disease through Induction of RANKL. Journal of dental research. 2015;94:968–75. 10.1177/0022034515577815 25808546

[21] Kursunlu SF, Ozturk VO, Han B, Atmaca H, Emingil G. Gingival crevicular fluid interleukin-36beta (-1F8), interleukin-36gamma (-1F9) and interleukin-33 (-1F11) levels in different periodontal disease. Archives of oral biology. 2015;60(1):77–83. 10.1016/j.archoralbio.2014.08.021 25247780

[22] Papathanasiou E, Teles F, Griffin T, Arguello E, Finkelman M, Hanley J, et al Gingival crevicular fluid levels of interferon-gamma, but not interleukin-4 or -33 or thymic stromal lymphopoietin, are increased in inflamed sites in patients with periodontal disease. Journal of periodontal research. 2014;49(1):55–61. 10.1111/jre.12078 23550893

[23] Saglam M, Koseoglu S, Aral CA, Savran L, Pekbagriyanik T, Cetinkaya A. Increased levels of interleukin-33 in gingival crevicular fluids of patients with chronic periodontitis. Odontology / the Society of the Nippon Dental University. 2016;Odontology. 10.1007/s10266-016-0259-0 27363844

[24] Tada H, Matsuyama T, Nishioka T, Hagiwara M, Kiyoura Y, Shimauchi H, et al Porphyromonas gingivalis Gingipain-Dependently Enhances IL-33 Production in Human Gingival Epithelial Cells. PLoS One. 2016;11(4):e0152794 10.1371/journal.pone.0152794 27058037 PMC4825981

[25] Saidi S, Bouri F, Lencel P, Duplomb L, Baud'huin M, Delplace S, et al IL-33 is expressed in human osteoblasts, but has no direct effect on bone remodeling. Cytokine. 2011;53(3):347–54. 10.1016/j.cyto.2010.11.021 21190867

[26] Kiyomiya H, Ariyoshi W, Okinaga T, Kaneuji T, Mitsugi S, Sakurai T, et al IL-33 inhibits RANKL-induced osteoclast formation through the regulation of Blimp-1 and IRF-8 expression. Biochemical and biophysical research communications. 2015;460(2):320–6. 10.1016/j.bbrc.2015.03.033 25795135

[27] Palmer G, Gabay C. Interleukin-33 biology with potential insights into human diseases. Nature reviews Rheumatology. 2011;7(6):321–9. 10.1038/nrrheum.2011.53 21519352

[28] Kawai T, Matsuyama T, Hosokawa Y, Makihira S, Seki M, Karimbux NY, et al B and T lymphocytes are the primary sources of RANKL in the bone resorptive lesion of periodontal disease. The American journal of pathology. 2006;169(3):987–98. 10.2353/ajpath.2006.060180 16936272 PMC1698808

[29] Liu D, Xu JK, Figliomeni L, Huang L, Pavlos NJ, Rogers M, et al Expression of RANKL and OPG mRNA in periodontal disease: possible involvement in bone destruction. International journal of molecular medicine. 2003;11(1):17–21. 12469211 10.3892/ijmm.11.1.17

[30] Usui M, Sato T, Yamamoto G, Okamatsu Y, Hanatani T, Moritani Y, et al Gingival epithelial cells support osteoclastogenesis by producing receptor activator of nuclear factor kappa B ligand via protein kinase A signaling. Journal of periodontal research. 2015;51(4):462–70. 10.1111/jre.12323 26432443

[31] Zhao JJ, Feng XP, Zhang XL, Le KY. Effect of Porphyromonas gingivalis and Lactobacillus acidophilus on secretion of IL1B, IL6, and IL8 by gingival epithelial cells. Inflammation. 2012;35(4):1330–7. 10.1007/s10753-012-9446-5 22382516

[32] Fujihara R, Usui M, Yamamoto G, Nishii K, Tsukamoto Y, Okamatsu Y, et al Tumor necrosis factor-alpha enhances RANKL expression in gingival epithelial cells via protein kinase A signaling. Journal of periodontal research. 2014;49(4):508–17. 10.1111/jre.12131 24102429

[33] Holtfreter B, Albandar JM, Dietrich T, Dye BA, Eaton KA, Eke PI, et al Standards for reporting chronic periodontitis prevalence and severity in epidemiologic studies: Proposed standards from the Joint EU/USA Periodontal Epidemiology Working Group. Journal of clinical periodontology. 2015;42(5):407–12. 10.1111/jcpe.12392 25808877 PMC7441325

[34] Saadi-Thiers K, Huck O, Simonis P, Tilly P, Fabre JE, Tenenbaum H, et al Periodontal and systemic responses in various mice models of experimental periodontitis: respective roles of inflammation duration and Porphyromonas gingivalis infection. Journal of periodontology. 2013;84(3):396–406. 10.1902/jop.2012.110540 22655910

[35] Sundnes O, Pietka W, Loos T, Sponheim J, Rankin AL, Pflanz S, et al Epidermal Expression and Regulation of Interleukin-33 during Homeostasis and Inflammation: Strong Species Differences. The Journal of investigative dermatology. 2015;135(7):1771–80. 10.1038/jid.2015.85 25739051

[36] Laperine O, Guicheux J, Lesclous P. Periostin-deficient mice, a relevant animal model to investigate periodontitis or not? BoneKEy reports. 2016;5:794 10.1038/bonekey.2016.21 27087940 PMC4822343

[37] Balato A, Di Caprio R, Canta L, Mattii M, Lembo S, Raimondo A, et al IL-33 is regulated by TNF-alpha in normal and psoriatic skin. Archives of dermatological research. 2014;306(3):299–304. 10.1007/s00403-014-1447-9 24522896

[38] Han X, Lin X, Yu X, Lin J, Kawai T, LaRosa KB, et al Porphyromonas gingivalis infection-associated periodontal bone resorption is dependent on receptor activator of NF-kappaB ligand. Infection and immunity. 2013;81(5):1502–9. 10.1128/IAI.00043-13 23439308 PMC3648000

[39] Lin J, Bi L, Yu X, Kawai T, Taubman MA, Shen B, et al Porphyromonas gingivalis Exacerbates Ligature-Induced, RANKL Dependent Alveolar Bone Resorption via Differential Regulation of Toll-Like Receptor 2 (TLR2) and TLR4. Infection and immunity. 2014;82:4127–34. 10.1128/IAI.02084-14 25047844 PMC4187858

